# Somatosensory Stimulus Intensity Encoding in Borderline Personality Disorder

**DOI:** 10.3389/fpsyg.2018.01853

**Published:** 2018-10-01

**Authors:** Kathrin Malejko, Dominik Neff, Rebecca C. Brown, Paul L. Plener, Martina Bonenberger, Birgit Abler, Georg Grön, Heiko Graf

**Affiliations:** ^1^Department of Psychiatry and Psychotherapy III, Ulm University, Ulm, Germany; ^2^Department of Child and Adolescent Psychiatry and Psychotherapy, Ulm University, Ulm, Germany; ^3^Department of Child and Adolescent Psychiatry, Medical University of Vienna, Vienna, Austria

**Keywords:** pain, parametric, borderline, fMRI, stimulus processing

## Abstract

Borderline Personality Disorder (BPD) is clinically characterized by emotional instability, interpersonal disturbances and dysfunctional behavior such as non-suicidal self-injury (NSSI). During NSSI, patients with BPD typically report analgesic or hypoalgesic phenomena, and pain perception and pain processing in BPD have been repeatedly investigated. Most of the studies so far focused on affective-motivational and cognitive-evaluative neural components of pain within categorial study designs. By contrast, rather basic somatosensory aspects such as neural intensity-encoding of somatosensory stimuli were not examined in further details. Thus, we investigated patients with BPD and healthy controls (HC) by functional magnetic resonance imaging (fMRI) during an unpleasant sensory stimulation task with parametrically increasing stimulus intensities. 15 females diagnosed with BPD and 15 HCs were investigated with fMRI during four individually adjusted levels of electrical stimulus intensities. Ratings of stimulus intensity were assessed by button presses during fMRI. fMRI-data were analyzed by analyses of variances (ANOVA) at a statistical threshold of *p* < 0.05 FWE-corrected on cluster level. Subjective ratings of stimulus intensities were alike between BPD and HC, and intensity levels identified with equal accuracy. Significant intensity-encoding neural activations were observed within the primary and secondary somtasensory cortex, the posterior insula, the posterior midcingulate cortex (pMCC) and the supplementary motor area (SMA) in both, HC and BPD. Notably, there were no significant between-groups differences in intensity-encoding neural activations, even at lowered significance thresholds. Present results suggest a similar neural somatosensory stimulus intensity encoding in BPD as previously observed on a behavioral level. The alterations in neural affective-motivational or cognitive-evaluative components reported so far may be restricted to pain rather than unpleasant stimulus processing and were absent in our study.

## Introduction

Borderline Personality Disorder (BPD) is a highly prevalent Cluster B personality disorder clinically characterized by emotional instability, interpersonal disturbances and dysfunctional behavior such as non-suicidal self-injury (NSSI) (Al-Alem and Omar, 2008). NSSI is often expressed as cutting or burning and suggested to serve as affect regulation by decreasing aversive inner tension (Shearer, 1994; Kleindienst et al., 2008). During NSSI, patients with BPD frequently report hypo- or analgesic phenomena (Leibenluft et al., 1987) and alterations in pain perception and pain processing were investigated in various studies (Schmahl et al., 2004, 2006; Schmahl and Bremner, 2006; Ludäscher et al., 2007; Niedtfeld et al., 2010, 2017).

Pain is considered as a nociceptive stimulus and as a fundamental sensory and affective state (Perl, 2007) and neuroimaging studies observed reliable neural activation within a broad network of brain regions (Iannetti and Mouraux, 2010). Whereas initial reports assumed a ‘pain-specific’ neural activation, more recent studies proposed that this network is not specifically activated by nociceptive stimuli rather than representing different submodalities of somatosensation (for review Iannetti and Mouraux, 2010). Thus, distinct but interacting subnetworks were described. The primary (SI) and secondary (SII) somatosensory cortex as well as the posterior insula are thought to encode sensory discriminative features (stimulus location, intensity and duration). In contrast, activation of the anterior insula and the anterior cingulate cortex (ACC) were assigned to the affective-motivational component (Davis, 2000; Peyron et al., 2000). Considering the distinct cingulate subdivisions (Vogt and Paxinos, 2014), anterior parts of the ACC such as the pregenual proportion (pgACC) are thought to encode affective responses (Vogt et al., 1996), whereas rather dorsal parts such as the dorsal anterior or midcingulate cortex (MCC) were associated with sensory features, cognitive and premotor functions in neural somatosensory processing (Büchel et al., 2002; Vogt, 2016; Tan et al., 2017).

Various studies mainly focused on pain perception in BPD and observed reduced pain sensitivity (Bohus et al., 2000; Schmahl et al., 2004, 2006; Ludäscher et al., 2007; Cárdenas-Morales et al., 2011). Neuroimaging studies demonstrated an altered neural pain processing in BPD with increased neural activation within the dorsolateral prefrontal cortex (dlPFC) but otherwise attenuated activation of the pgACC and of limbic regions such as the amygdala (Schmahl et al., 2006; Niedtfeld et al., 2010, 2017). This pattern was interpreted as a neuroanatomical proxy of an anti-nociceptive mechanism through downregulation of the emotional aspects of pain processing by increased top-down regulation. Based on this presumption of a changed affective appraisal of pain, subsequent studies mainly focused on the interaction of affect regulation (Niedtfeld et al., 2010) or social exclusion (Bungert et al., 2015) with pain processing, and on underlying functional connectivity in BPD (Kluestch et al., 2012; Niedtfeld et al., 2012).

However, rather somatosensory aspects such as the neural encoding of stimulus intensities have not yet been examined in detail in BPD, and most of the studies were conducted using particularly categorial designs by comparing neural activation under high versus low levels of painful stimuli (Schmahl et al., 2006; Niedtfeld et al., 2010, 2012, 2017; Kluestch et al., 2012). We therefore investigated patients with BPD and healthy controls (HC) by functional magnetic resonance imaging (fMRI) using a parametric study design with increasing levels of electrical stimulus intensities. Insofar, we planned the present study to bridge a gap between sensory extrema of pain processing and the encoding of basic sensory discriminative neural representation in BPD and specifically on neural correlates of parametrically increased stimulus intensities. The physiological neural encoding of stimulus intensity has been demonstrated by intensity-dependent activations within the SI, SII (Bornhövd et al., 2002; Valmunen et al., 2009; Grundmann et al., 2011; Case et al., 2016), the posterior insula (Ostrowsky, 2002; Geuter et al., 2017) and in dorsal subdivisions of the cingulate cortex (Büchel et al., 2002; Geuter et al., 2017). We expected disorder specific changes of these neural correlates considering reduced overall pain sensitivity in BPD (Bohus et al., 2000; Schmahl et al., 2004; Ludäscher et al., 2007).

## Materials and Methods

### Subjects

We investigated a clinical sample of 15 females diagnosed with BPD and 17 female HC matched for age and education within a case control design. The same sample was also investigated in a set of different experiment of which data will be reported elsewhere. After fMRI data acquisition, we detected fMRI artifacts in two subjects of the HC group that were then excluded from further analyses. Thus, our sample size in final analyses consisted of 15 patients diagnosed with BPD and 15 HC. A summary of the demographical data of patients included in final analyses is provided in **Table 1**. Patients with BPD were recruited from inpatient and outpatient units of the Department of Psychiatry and Psychotherapy of the Ulm University Hospital. HC were recruited from the campus of Ulm University. All participants gave written informed consent prior to the study that was approved by the local ethical committee of Ulm University and conducted in accordance with the Declaration of Helsinki.

**Table 1:** Summary of demographical data and psychometric measures in patients with borderline personality disorder (BPD; *n* = 15) and healthy controls (HC; *n* = 15).

	HC	BPD	*t*-test	
	**mean (sem)**	**mean (sem)**	***t* (2,28)**	***p***
*Demographical data*				
Age (years)	23.27 (1.11)	23.33 (1.07)	−0.04	0.966
Education (school years)	10.60 (0.40)	10.47 (0.70)	0.23	0.821
*Psychometric measures*				
BDI	4.80 (1.45)	40.07 (3.54)	−9.22	*0.000*
BSL	0.23 (0.07)	2.45 (0.33)	−6.53	*0.000*
BIS	59.87 (1.94)	70.00 (2.20)	−3.46	*0.002*

*Statistical analyses were conducted by two-sided unpaired t-tests. Significant (p < 0.05) p-values are in italic font. BDI, Beck Depression Inventory; BSL, Borderline Symptom List; BIS, Barratt Impulsiveness Scale; sem, standard error of the mean; n, number of subjects.*

Participants with any severe medical, substance use, or psychotic disorders were excluded from the study. Exclusion criteria for HC were any current or lifetime psychiatric disorder. In the BPD group, 13 patients were diagnosed with a comorbid major depressive disorder; in the two remaining patients, depression was remitted at the time of investigation. Moreover, 8 patients with BPD also met criteria of posttraumatic stress disorder, and 2 further patients met criteria of dysthymia according to DSM-IV. Psychopharmacological treatment in patients with BPD was not interrupted except of sedative medication prior to fMRI scanning. In detail, 13 patients with BPD took antidepressant medication (2 fluoxetine, 1 trimipramine and venlafaxine, 1 fluoxetine and mirtazapine, 1 tranylcypromine and lithium, 1 sertraline and mirtazapine and doxepine, 1 sertraline and mirtazapine, 1 sertraline, 1 escitalopram, 1 mirtazapine, 1 venlafaxine, 1 sertraline and bupropion, 1 bupropion). None of the subjects in the BPD-group took any other medication other than mentioned, and none of the subjects in the HC-group took any medication. Notably, none of the subjects of either group took analgesic medication, neither temporarily nor continuously.

### Psychometric Measurements

Current or past psychiatric disorders were assessed by one of the study psychologists or physicians using the Structured Clinical Interview for DSM-IV (SCID-I and -II; First et al., 1997). Symptom severity in BPD was assessed by the Borderline Symptom List (BSL; Bohus et al., 2007) and current depressive symptoms were examined by the Beck Depression Inventory (second edition, BDI-II; Beck et al., 1996) in its German version (Hautzinger et al., 2006). To assess impulsivity as personality trait we applied the Barratt Impulsiveness Scale in its 11th revision (BIS-11; Barratt, 1994; Patton et al., 1995). The BIS-11 is a self-reporting questionnaire that contains 30 items, each rated on a 4-point Likert scale ranging from 1 (rarely/never) to 4 (almost always). Thus, higher sum-scores reflect higher trait impulsivity. Two-sided unpaired *t*-tests were computed to analyze demographical data and psychometric scales at a statistical significance level of *p* < 0.05 (see **Table 1**). To examine whether patients with BPD were engaged in actual self-injurious behavior, self-mutilation was assessed by the Functional Assessment of Self-Mutilation (FASM) (Lloyd, 1997) in its German version (Kaess et al., 2013). The corresponding results are provided in our **Supplementary Material** (**Supplementary Table S1**).

### fMRI Experimental Task

During fMRI we used an established scalable electric stimulation equipment that has been shown to induce unpleasant (but not painful) somatosensation (Adolph et al., 2010; Cárdenas-Morales et al., 2011; Bonenberger et al., 2015a,b) Somatosensory unpleasant stimuli during fMRI were applied via electric stimulation in 4 parametrically increasing levels of intensity over the dorsum of the left, non-dominant hand. Electrical stimulation was applied on entirely intact skin. One stimulus consisted of a train of four electrical square pulses with duration of 1 ms each (500 Hz). The train was delivered at a frequency of 20 Hz for 1 s by a constant current stimulator that was built into a conventional device for electromyographic investigations (Medtronic Dantec Keypoint portable, Skovlunde, Denmark). The experimental stimuli were conform to the guidelines for experimental pain (non-invasive, no tissue damage, avoiding movement, ethically acceptable, reproducible, and physiologically relevant) (Petersen-Felix and Arendt-Nielsen, 2002). Subjects rated stimulus intensity by button presses on a four-button box in their right hand and were instructed by speed versus accuracy. To control for effects of expectation and attention, a signal tone was presented 1.5 s before applying the electrical stimuli. A total of 24 electrical stimuli (6 per level) were applied in a pseudo-randomized order during the fMRI scan. The interstimulus interval between stimuli of the same intensity was 24.3 s. Entire duration was 10 min and 43 s for the whole task.

Individual upper and lower boundaries of the 4 stimulus intensity levels were assessed prior to fMRI-scanning. In detail, the minimum stimulus intensity was assessed as the lowest level the subject reliably perceived the stimulus (defined as level 1). Subjects gave direct feedback and permission to increase stimulus intensity (after each single step). Current amplitude could be varied in steps of 0.1 mA. Intensity of the electrical stimulus was then increased stepwise to the individual maximum intensity that was perceived as unpleasant but not painful (defined as level 4) such that subjects were willing to experience this intensity level repeatedly. Intensity levels 2 and 3 were spaced equidistantly in-between levels 1 and 4. After the individual assessment, subjects were trained to correctly rate the stimulus levels 1 to 4 with stimuli provided in randomized order.

Due to technical conditions, we had to use different stimulus electrodes for 4 participants of the HC and for the whole BPD group that restricted the direct comparison of electrical stimulus intensity levels between groups (see **Supplementary Material** for details on stimulus electrodes). Thus, ratios of maximum (level 4) and minimum stimulus intensities (level 1) were computed and served for group comparisons. Two-sided *t*-tests (*p* < 0.05) were calculated to analyze accuracy of stimulus ratings and the ratio of stimulus intensity levels.

### fMRI Data Acquisition

Functional imaging data were obtained by a 3T MAGNETOM Allegra (Siemens, Erlangen, Germany). High resolution anatomical T1-weighted images (1 × 1 × 1 mm voxels) were obtained (bandwidth (BW) = 130 Hz/Pixel, repetition time (TR) = 2500 ms, inversion time (TI) = 1.1 s, echo time (TE) = 4.57 ms, flip angle = 12°). T2*^∗^*-weighted functional MR images were obtained using gradient echo-planar imaging sequences. 35 transversal slices were recorded at a TR of 2000 ms with an image size of 64 × 64 pixels, a field of view (FOV) with 230 mm, a slice thickness of 2.5 mm and an interslice gap of 0.5 mm. TE was 33 ms with a flip angle of 90°. In total, 305 volumes were recorded during the sensory stimulation task. The first 6 volumes were discarded from further analyses to allow for T1 equilibration.

### fMRI Data Analysis

Image preprocessing and statistical analyses we carried out by using Statistical Parametric Mapping (SPM12, Wellcome Department, London, United Kingdom) with a random effects model for group analyses. Data were preprocessed including slice time correction, realignment, and normalization into a standard template (Montreal Neurological Institute, MNI). Smoothing was applied with an 8- mm FWHM isotropic Gaussian kernel. Intrinsic autocorrelations were accounted for by AR(1) and low frequency drifts were removed via high-pass filtering.

For individual first level analyses, a general linear model was used to estimate the height of neural activation associated with each of the four stimulus intensities. Onsets of individual trials for each of the four different intensity levels were modeled as stick functions and were convolved with the hemodynamic response function. Regressors representing the 6 motion parameters were added to the design matrix in addition and were integrated into the statistical design as were the onsets of the preceding warning tone and motor responses as regressors of no interest.

For second level group analysis, we computed analyses of variances (ANOVA) with the factors ‘group’ (HC, BPD) and ‘condition’ (stimulus intensity levels) including first level contrast images for each stimulus intensity level. A main effect ‘condition’ and a ‘group’-by-‘condition’ interaction effect were modeled. One tailed directed t-contrasts were conducted to test for differential parametric effects (increasing stimulus intensity levels) in BPD and HC separately. Between group differences of parametric increases were tested on significance with one tailed t-contrasts (HC > BPD; HC < BPD). Also, a conjunction analyses was computed to examine conjointly significant clusters of increasing neural activation in BPD and HC. Statistical inference levels were the same as for all analyses above: *p* < 0.001, uncorrected at the voxel level in combination with a minimum cluster size of 167 contiguously significant voxels to infer significant clusters at a level of *p* < 0.05 for FWE-correction at the cluster level.

To verify our results from fMRI, we computed further analyses that are summarized in the **Supplementary Material** section: To summarize here, we controlled for medication and conducted a sub-group analyses between the 13 patients with BPD that were on antidepressive medication and HC (see **Supplementary Table S2**). Second, we considered differences in impulsiveness as measured by the BIS for between-group analyses, and conducted a two-sample *t*-test including the individual values of this scale as a covariate. Third, taking into account the previous neural alterations in pain processing within the dlPFC and the pgACC in BPD (Schmahl et al., 2006), we extracted parameter estimates from clusters comprising peak voxel activation as provided by Schmahl et al. (2006) surrounded by a 5 mm sphere (see **Supplementary Figure S1**). At least, we conducted power analyses from these data to estimate the sample sizes that would be necessary to keep the null hypothesis with second order error probability of beta=*p* < 0.20 (i.e., power of 0.8).

## Results

### Demographical and Behavioral Data

In line with the clinical diagnosis, significant higher sum-scores in the BSL and BIS were observed in patients with BPD compared to HC. Furthermore, patients with BPD showed higher BDI-II ratings relative to HC. Mean total scores of the psychometric measurements, as well as *t*- and *p*-values associated with group comparisons are summarized in **Table 1**. Regarding the behavioral responses in our stimulation task during fMRI, patients with BPD and HC rated stimulus intensity levels with equal accuracy (false responses: BPD mean = 8.40, sem = 0.83; HC mean = 8.87, sem = 0.80; *t*(2,28) = 0.41; *p* = 0.688). However, a trend toward a slightly higher ratio of stimulus intensity was observed in BPD compared to HC (Maximum unpleasant intensity level / minimum unpleasant intensity level: BPD mean = 7.00, sem = 1.04, HC mean = 4.42, sem = 0.71; *t*(2,28) = −2.04; *p* = 0.050).

### fMRI Data

**FIGURE 1:**
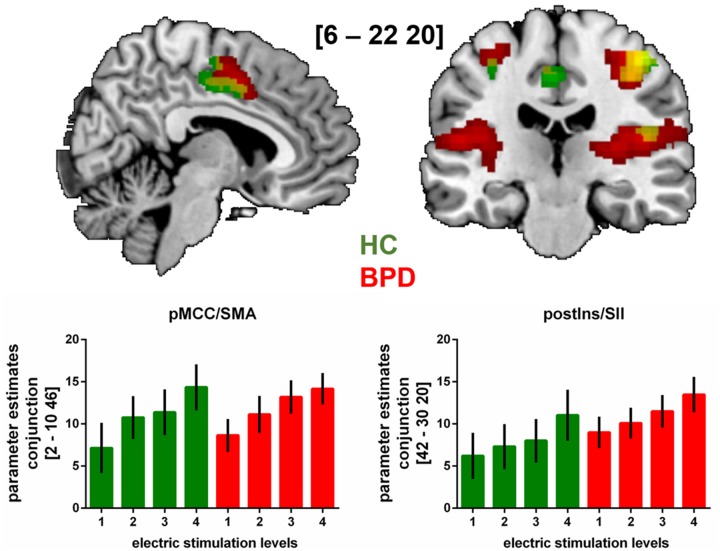
fMRI-results from whole brain analysis of neural activation during the sensory stimulation task with increasing stimulus intensities (levels 1 to 4) in patients with BPD and HC.

Regarding neural activation encoding increasing stimulus intensities, our whole brain analysis observed a significant (*p* < 0.05 FWE-corrected) neural response pattern within the bilateral primary somtasensory cortex (SI), right secondary somatosensory cortex (SII) and posterior and middle insula as well as in right posterior midcingulate cortex (pMCC) and supplementary motor area (SMA) in HC. In BPD-patients, neural activation within bilateral SI and SII, bilateral posterior and left middle insula, pMCC/SMA and cerebellum corresponded significantly to increasing stimulus intensity at the same statistical threshold as in HC. A conjunction analysis revaeled conjontly significantly increasing neural activation within right SI, SII, posterior insula and right pMCC/SMA with increasing sensory stimulus intensities in both groups (see **Figure 1** and **Table 2**). Between-group comparisons revealed no significant differences in differential neural activation between BPD and HC, even when abandoning family-wise error correction at the cluster level. The corresponding BOLD-signal time courses are provided in our **Supplementary Material** (see **Supplementary Figure S2**). Results from sub-group analyses (see **Supplementary Table S2**) and between-group comparison including BIS as covariates are also provided in our **Supplementary Materials**. Briefly, also in the presence of this covariate, no significant between-group differences emerged.

**Table 2:** Significant (*p* < 0.05; FWE-corrected on cluster-level) neural activation in whole brain analysis corresponding to increasing sensory stimulus intensities in HC (*n* = 15) and patients with borderline personality disorder (BPD; *n* = 15).

	*BA*	*Anatomic*	*L/R*	*Cluster size*	*Z*	*MNI*		
		*Label*		*k (Vx)*		*x*	*y*	*z*
*HC*	23	pMCC/SMA	R	502	4.93	0	−18	46
					3.78	4	−4	42
					3.76	2	−10	56
	3	SI	R	472	5.86	44	−22	56
	4	SI	L	216	4.05	−34	−26	52
					3.94	−40	−28	62
	48	postIns/SII	R	209	3.99	42	−30	20
					3.95	48	−22	16
					3.58	42	−16	18
	48	Middle insula	R	189	4.79	56	12	2
					3.58	60	−2	8
					3.30	48	2	2
*BPD*	24	pMCC/SMA	R	1276	5.15	2	−6	50
					5.06	2	4	46
					4.78	−6	10	38
	3	SI	R	1152	6.29	40	−20	58
					6.10	40	−20	46
					4.00	56	−14	40
	3	SI	L	306	4.81	−36	−24	56
					4.74	−26	−28	62
	48	postIns/SII	R	1905	5.59	42	−18	14
					5.26	46	−32	22
					5.00	32	−22	10
	48	postIns/SII	L	1341	5.70	−50	−24	14
					4.97	−38	−20	14
					4.82	−46	−36	22
	48	Middle insula	L	196	5.29	−54	2	0
	37	Cerebellum	L	207	4.53	−18	−54	−20
					3.58	−34	−54	−28
*Conjunction*	24	pMCC/SMA	R	295	4.18	2	−10	46
					3.94	2	−10	56
					3.89	4	2	40
	3	SI	R	413	6.53	44	−22	56
	48	postIns/SII	R	202	4.20	42	−30	20
					4.15	48	−22	16
					3.74	42	−16	18

*BA, Brodman area; L, left; R, right; k, number of voxels (Vx); MNI, Montreal Neurological Insitute (x-, y-, z-coordinates are provided in mm); z, z-score of standard norm distribution; pMCC, posterior midcingulate cortex; SMA, supplementary motor area; SI, primary somatosensory cortex; SII, secondary somatosensory cortex; postIns, posterior insula; n, number of subjects.*

Brain slides depict significant (*p* < 0.05 FWE-corrected on cluster level) neural activation under increased sensory stimulus intensities in BPD, HC and in both groups (conjunction analyses). Bar charts show fMRI parameter estimates extracted from peak voxel activation in the conjunction analyses within the pMCC/supplementary motor area (SMA) and the posterior insula/secondary somatosensory cortex (postIns/SII) with standard errors of the mean. MNI-coordinates [*x*,*y*,*z*] are provided in mm.

## Discussion

We investigated patients with BPD and HC using fMRI to specifically investigate neural activation encoding the somatosensory discrimination of increasing unpleasant electrical stimulus intensities. Behavioral results during the sensory electrical stimulation task demonstrated, that both, patients with BPD and HC rated intensity levels with equal accuracy. However, slightly higher ratios of stimulus intensities had to be applied in BPD. During fMRI, neural activation within bilateral SI, right SII, posterior and middle insula as well as in right pMCC/SMA increased as a function of stimulus intensity in HC. Likewise, in patients with BPD, neural activation increased with elevated intensities within bilateral SI and SII, bilateral posterior and the left middle insula, pMCC/SMA and cerebellum. A conjunction analysis confirmed that the neural activation pattern within right SI, SII, posterior insula and right pMCC/SMA was alike in both groups. Reliable between-group differences were not to observe.

In line with the diagnoses of our clinical sample, we found higher levels of impulsivity as personality trait and higher borderline symptom severity in patients with BPD compared to HC. Self-rated depressive symptoms were more frequent in the BPD group and in line with the well-known high comorbidity between depression and BPD (Distel et al., 2016). In our fMRI sensory electrical stimulation task, we observed a trend to a higher ratio of the maximum to minimum stimulus intensity in patients with BPD compared to HC. The higher ratio may refer to the reduced pain sensitivity regarding experimental pain as previously observed in BPD (Bohus et al., 2000; Ludäscher et al., 2007). Notably, this conclusion has to be regarded with caution considering that in our study two different electrodes were used within the HC group but also between groups. This critically restricted comparability, but did not interfere with our purpose to investigate the discrimination of different stimulus intensity levels rather than pain perception. Patients with BPD and HC rated the stimulus intensity levels with equal accuracy. This is in line with a previous investigation in BPD conducted with a similar sensory stimulation task with parametrically increasing levels of intensities (Cárdenas-Morales et al., 2011). Moreover, this result is in accordance with the assumption that patients with BPD do not reveal generalized somatosensory deficits (Pavony and Lenzenweger, 2013).

An unimpaired sensory discrimination of unpleasant sensory stimulus intensity in BPD is not only supported by our behavioral results, but also by similar neural activation while encoding stimulus intensity compared to HC. Parallel to increasing stimulus levels, we observed an increase in neural activation within SI and SII. The contribution of these regions to neural intensity coding has been consistently demonstrated (Bornhövd et al., 2002; Valmunen et al., 2009; Grundmann et al., 2011; Case et al., 2016). Other intensity-encoding neural activation were observed within the posterior insula. This pattern of activation is supported by a recent fMRI study conducted with model-based analyses that underpinned the stimulus intensity-encoding role of the posterior insula also regarding innocuous stimuli as used in our study (Ostrowsky, 2002; Geuter et al., 2017). It is further in line with the functional posterior to anterior gradient within the insular cortex due to its cytoarchitectonic organization (Cerliani et al., 2012; Nieuwenhuys, 2012). According to this theory, rather posterior parts encode somatosensory features (Nieuwenhuys, 2012) owing to the direct spinothalamic input (Dum et al., 2009) and the functional and structural connection to somatosensory cortices (Wiech et al., 2014) of this subdivision.

Neural stimulus intensity scaling was further observed in the cingulate cortex in both, patients with BPD and HC. While ventral parts of the cingulate cortex such as the pgACC are related to affective coding (Vogt et al., 1996) and thought to be decreased in BPD (Schmahl et al., 2006; Niedtfeld et al., 2010, 2017), rather dorsal subdivisions are most likely associated with somatosensory stimulus processing (Büchel et al., 2002; Vogt, 2016; Tan et al., 2017). Specifically, neural activation within the pMCC as found in our study has been assigned to noxious but also innocuous cutaneous stimulation (Vogt, 2016) and align with stimulus intensity (Büchel et al., 2002; Geuter et al., 2017). Together with the SMA, this activation may contribute to a neural control mechanism over reflexive motor activity (Vogt, 2016).

Despite the finding that intensity-encoding neural activation was more extended in BPD than in HC, it is of note that we found no significant differences in comparison to HC. Only within the cerebellum, a neural intensity scaling was found in BPD but not in HC. Although the stimulus encoding role of the cerebellum has been previously described (Helmchen et al., 2003), a disorder-specific alteration in cerebellar activation in BPD cannot be infered due to the lack of significant group differences. Furthermore, neural activation in this region owing to stimulus intensity was also found in HC under a more lenient statistical threshold of *p* < 0.001, with no cluster-size correction for multiple comparisons.

Thus, our results lend further support for a similar behavioral discrimination and neural encoding of somatosensory stimulus intensities in BPD compared to HC. This is in line with previous investigations finding unimpaired exteroception (Pavony and Lenzenweger, 2013) and similar discrimation of aversive stimuli intensities in BPD compared to HC (Cárdenas-Morales et al., 2011) on a behavioral level. Present lack of neural alterations in cerebral networks linked to the affective component of pain as found in other studies in BPD (Schmahl et al., 2006; Niedtfeld et al., 2017) may be owed to the fact that our clinical sample was investigated under antidepressant medication. Apart from the antinociceptive effects of antidepressants (Singh et al., 2001; Sikka et al., 2011), their impact on brain activations especially on brain regions contributing to the affective component of somatosensory stimulus processing such as the pgACC or the amygdala, has been well established (Wessa and Lois, 2015). This interpretation is supported when taking into account that alterations in affective-motivational components of cerebral networks processing pain under painful stimulation in BPD were observed in the absence of medication (Schmahl et al., 2006; Ludäscher, 2010; Niedtfeld et al., 2010, 2017). Also, the lack of alterations in affective-motivational components in the present BPD sample compared to HC may rely on our study design considering that we applied unpleasant but not painful stimulus intensities. One could assume that a further increase of intensity levels up to painful stimulation may exceed a threshold with regard to saliency, that initiates the previously described alterations in affective appraisal in BPD (Schmahl et al., 2006; Niedtfeld et al., 2010, 2017). However, this remains speculative and to examine this issue would require a study design including increasing levels of innocuous but also noxious, painful stimuli. Another limitation arises by the comorbid depressive disorder in our group of BPD patients. A current meta-analysis assumes that variable and multiple factors contribute to the interaction of depressive disorder and pain perception (Thompson et al., 2016) but results from neuroimaging studies are scarce (Bär et al., 2007; Strigo et al., 2008, 2013). Nonetheless, it seems unlikely that the comorbid depression drove the approximation of neural intensity-encoding in BPD toward that in HC.

Due to the experimental setup, the current dissociative or emotional state immediately prior to the electrical stimulation were not assessed in BPD in our study. This may represent a limitation considering their impact on pain sensitivity and on neural pain processing network (Ludäscher, 2010; Orenius et al., 2017). Moreover, our study was conducted with a relatively small sample size considering that well-characterized patients with BPD without comorbid psychiatric disorder other than depression are hard to acquire. This compromises the generalizability of our data but power analyses (see **Supplementary Material**) revealed that approximately 55 to 60 participants in each group were necessary to keep the null hypothesis with an error rate of at least 0.2. Thus, our results await empirical replication with larger samples.

In summary, we could demonstrate stimulus intensity encoding neural activations within the primary and secondary somatosensory cortex, the posterior insula and the pMCC and adjacent the SMA in BPD which appear to be alike to those in HC. Thus, our study may lend further support for unimpaired basic somatosensory stimulus processing as already observed on a behavioral level (Cárdenas-Morales et al., 2011). The alterations in neural networks related to affective-motivational or cognitive-evaluative components of pain processing previously described in BPD may have been ameliorated by antidepressant medication or, alternatively or complementary, may be restricted to the neural processing of pain rather than unpleasant, innocuous sensory stimuli as used in our study.

## Author Contributions

HG and KM contributed substantially to the work, analyzed and interpreted the data, and drafted the manuscript. BA, RB, GG, and PP contributed substantially to the work, interpreted the data, and revised the manuscript critically for important intellectual content. MB, DN, and RB obtained the data. All the authors approved the final version to be published and agreed to be accountable for all aspects of the work in ensuring that questions related to the accuracy or integrity of any part of the work are appropriately investigated and resolved.

## Conflict of Interest Statement

The authors declare that the research was conducted in the absence of any commercial or financial relationships that could be construed as a potential conflict of interest.
