# Supplementary material for: Somatosensory Stimulus Intensity Encoding in Borderline Personality Disorder
Source: Front Psychol. 2018 Oct 1;9:1853. doi: 10.3389/fpsyg.2018.01853 (PMC6174222; doi:10.3389/fpsyg.2018.01853)
Supplement: Supplementary file 1 [file Data_Sheet_1.docx]

**Supplementary Material**

**Somatosensory stimulus intensity encoding in borderline personality disorder**

Kathrin Malejko^1*^, Dominik Neff^1^, Rebecca C Brown^2^, Paul L Plener ^2,3^, Martina Bonenberger^2^, Birgit Abler^1^, Georg Grön^1^, Heiko Graf^1^

^1^ Ulm University, Department of Psychiatry and Psychotherapy III, Ulm, Germany

^2^ Ulm University, Department of Child and Adolescent Psychiatry and Psychotherapy, Ulm, Germany

^3^ Medical University Vienna, Department of Child and Adolescent Psychiatry, Vienna, Austria

**METHODS**

**Details on stimulus electrodes**

For technical reasons, we had to use different stimulus electrodes for 4 participants of the HC and for the whole BPD group. In detail, we used an universal size standard grounding pad (Ambu® Australia Pty Limited; Neutralect 2405M) for 11 subjects of the HC-group. For 3 subjects of the HCs and for all patients with BPD, we used an universal size split grounding pad (Ambu® Australia Pty Limited; Neutralect 2406M). When using the Neutralect 2406M pad, we had to apply higher amplitudes to overcome perception thresholds, presumably due to the split (within one grounding pad) and an increase in electrical resistance. This restricted a direct comparison between electric currents or amplitudes.

**RESULTS**

**Psychometric measurements on Self-Mutilation as assessed by the Functional Assessment of Self-Mutilation (FASM) scale**

**Table S1:** Severe and minor non-suicidal self-injury (NSSI) of 14 subjects of the BPD-group within the last 12 months. Data from one subject with BPD were not recorded. n=number of subjects.

|  |  |  | |
| --- | --- | --- | --- |
| **type of NSSI** | | **n** | **% (of n=14)** |
| *Severe NSSI* | |  |  |
|  | cut/carved on skin | 12 | 86% |
|  | scraped skin | 10 | 71% |
|  | erased skin | 7 | 50% |
|  | burned skin | 4 | 29% |
| *Minor NSSI* | |  |  |
|  | picked at a wound | 14 | 100% |
|  | bit itself | 13 | 93% |
|  | hit self on purpose | 8 | 57% |
|  | picked at areas of body to draw blood | 4 | 29% |
|  | Pulled hair out | 3 | 21% |
|  | Inserted objects under nails or skin | 1 | 7% |
| *Others* | |  |  |
|  | Suicide attempt | 2 | 14% |
|  | Starve/vomit | 2 | 14% |
|  | Drug use | 1 | 7% |
|  | Use of ice pack until frostbite | 1 | 7% |
|  | Hit a wall on purpose | 1 | 7% |
|  | Pulled toenails out | 1 | 7% |

**fMRI sub-group analysis comparing HCs and the 13 patients diagnosed with BPD that took psychopharmacological (antidepressant) medication**

|  | *BA* | *Anatomic* | *L/R* | *cluster size* | *Z* | *MNI* | | |
| --- | --- | --- | --- | --- | --- | --- | --- | --- |
|  |  | *label* |  | *k (Vx)* |  | *x* | *y* | *z* |
| *HC* | 23 | pMCC/SMA | R | 499 | 4.89 | 0 | -18 | 46 |
|  |  |  |  |  | 3.98 | 4 | -8 | 44 |
|  |  |  |  |  | 3.77 | 4 | 2 | 40 |
|  | 3 | SI | R | 472 | 5.91 | 44 | -22 | 56 |
|  | 4 | SI | L | 193 | 3.99 | -34 | -26 | 52 |
|  |  |  |  |  | 3.89 | -40 | -28 | 62 |
|  | 48 | postIns/SII | R | 232 | 3.99 | 42 | -30 | 20 |
|  |  |  |  |  | 3.95 | 48 | -22 | 16 |
|  |  |  |  |  | 3.53 | 42 | -16 | 18 |
|  | 48 | middle insula | R | 197 | 4.75 | 56 | 12 | 2 |
|  |  |  |  |  | 3.55 | 60 | -2 | 8 |
|  |  |  |  |  | 3.40 | 50 | 2 | 4 |
| *BPD* | 24 | pMCC/SMA | R | 1006 | 4.75 | 2 | -6 | 50 |
|  |  |  |  |  | 4.62 | 2 | 4 | 46 |
|  |  |  |  |  | 3.88 | 12 | 10 | 44 |
|  | 3 | SI | R | 857 | 5.84 | 40 | -20 | 58 |
|  |  |  |  |  | 5.32 | 40 | -20 | 46 |
|  |  |  |  |  | 4.38 | 42 | -10 | 58 |
|  | 3 | SI | L | 364 | 5.07 | -36 | -26 | 62 |
|  |  |  |  |  | 3.94 | -26 | -42 | 54 |
|  | 48 | postIns/SII | R | 1529 | 5.25 | 42 | -20 | 14 |
|  |  |  |  |  | 5.05 | 30 | -20 | 10 |
|  |  |  |  |  | 4.87 | 60 | -28 | 20 |
|  | 48 | postIns/SII | L | 1418 | 5.30 | -50 | -24 | 14 |
|  |  |  |  |  | 4.66 | -46 | -36 | 22 |
|  |  |  |  |  | 4.59 | -38 | 0 | 12 |
|  | 48 | middle insula | L | 154 | 4.79 | -54 | 2 | 2 |
|  | 48 | middle insula | R | 183 | 4.38 | 54 | 2 | 4 |
| *conjunction* | 24 | pMCC/SMA | R | 243 | 3.85 | 4 | -12 | 46 |
|  |  |  |  |  | 3.72 | 2 | -8 | 54 |
|  |  |  |  |  | 3.71 | 4 | 2 | 42 |
|  | 3 | SI | R | 394 | 5.51 | 42 | -22 | 56 |
|  | 48 | postIns/SII | R | 214 | 3.99 | 42 | -30 | 20 |
|  |  |  |  |  | 3.95 | 48 | -22 | 16 |
|  |  |  |  |  | 3.53 | 42 | -16 | 18 |

**Table S2:** Significant (p<0.05; FWE-corrected on cluster-level) neural activation in whole brain analysis corresponding to increasing sensory stimulus intensities in healthy controls (HC; n=15) and patients with borderline personality disorder (BPD; n=13):

BA=Brodman area; L=left; R=right; k=number of voxels (Vx); MNI=Montreal Neurological Insitute (x-, y-, z-coordinates are provided in mm), z=z-score of standard norm distribution;; pMCC=posterior midcingulate cortex; SMA=supplementary motor area; SI=primary somatosensory cortex; SII=secondary somatosensory cortex; postIns=posterior insula; n=number of subjects.

**Power analyses and parameter estimates from the dorsolateral prefrontal cortex (dlPFC) and the pregenual anterior cingulate cortex (pgACC)**

As mentioned in our introduction, neuroimaging studies demonstrated an altered neural pain processing in BPD with increased neural activation within the dorsolateral prefrontal cortex (dlPFC) but otherwise attenuated activation of the pgACC (Schmahl et al., 2006). This pattern was interpreted as a neuroanatomical proxy of an anti-nociceptive mechanism through downregulation of the emotional aspects of pain processing by increased top-down regulation.

To examine neural activations within these regions, we extracted differential (high versus low; level 4 minus level 1 in each group) parameter estimates from the cluster comprising peak voxel activations (left dlPFC x/y/z in mm: -48/-26/20; pgACC x/y/z in mm: -1/25/1) as provided by Schmahl et al. (2006) and surrounded by a 5 mm sphere.

As depicted in **Figure S1**, we found a similar neural response pattern with a numerical increase in neural activations within the dlPFC and attenuated pgACC activations in BPD compared to HCs. However, statistical inference revealed no significant differences between BPD and HC, neither within the dlPFC (t(2,28)=1.02; p=0.317) nor within the pgACC (t(2,28)=-0.73; p=0.472).

**
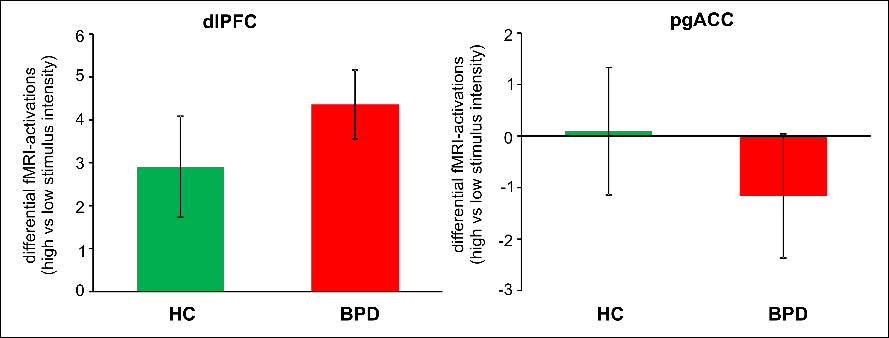
**

**Figure S1:** Differential (high versus low stimulus intensity) neural activations within the left dorsolateral prefrontal cortex (dlPFC; upper panel) and the left pregenual anterior cingulate cortex (pgACC; lower panel) in BPD (n=15; red) and HC (n=15; green). Bar graphs with standard error of the mean depicts differential parameter estimates in high (stimulus intensity level 4) versus low (stimulus intensity level 1) stimulus intensity, extracted from a cluster comprising peak voxel activations as provided by Schmahl et al. 2006 (surrounded by a 5 mm sphere).

From these data we also computed post-hoc power analyses (G*Power Version 3.1.9.2) and, as expected, with a sample size of 15 subjects in each group, post-hoc power was 0.54 for the non-difference in dlPFC, and 0.58 in pgACC, with corresponding error rates for keeping the null-hypothesis of beta = 0.46 in case of dlPFC and beta = 0.42 in case of pgACC.

We also calculated power analyses from that data, to estimate the sample sizes necessary to keep the null hypothesis with an error rate of at least 0.2, which resulted in an estimate of around 55 to 60 participants in each group. Thus, the absence of group differences in our study is to be treated with caution since for keeping the null hypothesis actual sample sizes were certainly underpowered.

Alternatively, pain is considered as a nociceptive stimulus and as a fundamental sensory and affective state (Perl, 2007). A corollary of this is that pain must have a (i.e. emotional) quality other than unpleasantness. Thus, neural alterations within the dlPFC or the pgACC may not be evident considering their contribution to cognitive and affective domains of somatosensation.

**BOLD-signal time courses**

**Figure S2:** BOLD signal time courses

The raw BOLD-signal time courses were extracted from the right primary somatosensory cortex (SI), the cluster comprising the posterior midcingulate cortex (pMCC) and the supplementary motor area (SMA) as well as from the cluster comprising the posterior insula (postIns) and the secondary somatsosensory cortex (SII) from healthy controls (HC) and patients with borderline personality disorder (BPD). BOLD-signal time courses with standard error of the mean (sem) for the different stimulus intensity levels (level 1 to 4) are depicted in colors (HC: dark to lime green; BPD: dark red to pink). x-axis: seconds (TR=2000ms).


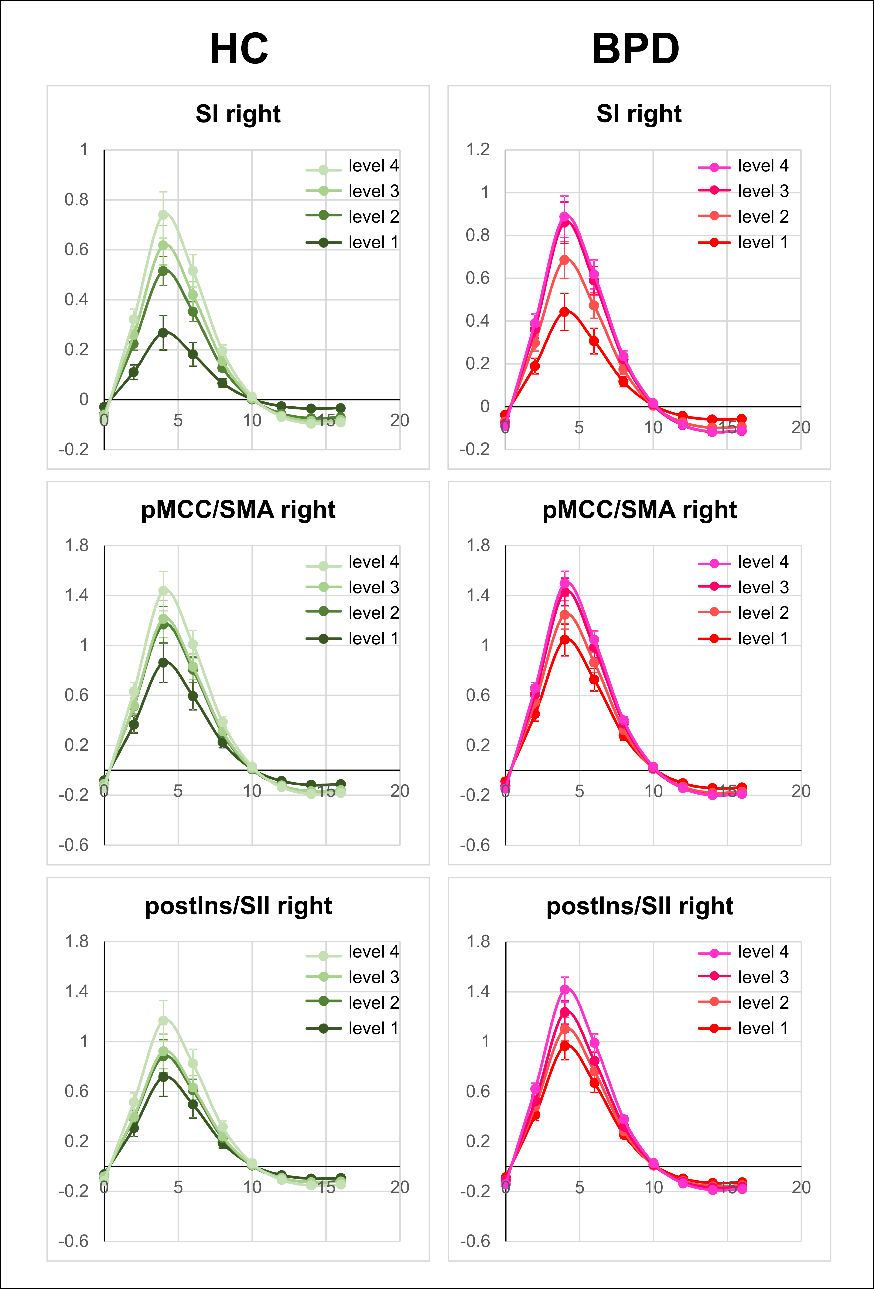


**Two-sample t-tests with psychometric measures as covariate**

To control for the differences on psychometric measures (BDI, BSL and BIS) between groups (HC and BPD), we further conducted two-sample t-tests, each including the individual values of these scales as covariate. However, we found no significant (p<0.05 FWE-corrected on cluster level) differences in neural intensity encoding activations between BPD and HC by these analyses.

**References**

Perl, E. R. (2007). Ideas about pain, a historical view. *Nat Rev Neurosci* 8, 71–80. doi:10.1038/nrn2042.

Schmahl, C., Bohus, M., Esposito, F., Treede, R.-D., Di Salle, F., Greffrath, W., et al. (2006). Neural correlates of antinociception in borderline personality disorder. *Arch. Gen. Psychiatry* 63, 659–667. Available at: http://www.scopus.com/inward/record.url?eid=2-s2.0-33744943318&partnerID=40&md5=781d4fe2603415869bf7d5e4991d69c7.
